# The Evolution of Medical Societies in Britain - Have They a Future?
*The substance of the presidential address given on 10 October 1979. Because of late publication of the Journal its cover date antecedes the giving of this address.


**Published:** 1979

**Authors:** Donald S. Ractliffe


### The Evolution of Medical Societies in Britain – Have They a Future?*

**Donald S. Ractliffe**
General Practitioner

Five years ago, in the Society's centenary year, Professor Bruce Perry gave a scholarly address on 'The History of Medicine in Bristol and of the Bristol Medico-Chirurgical Society' which he said 'for the last hundred years had been really synonymous' and he ended 'by congratulating the Society on the success of its first hundred years and wishing it an even more successful future.'[1]

I too wish it a successful future but we need to give some thought to the matter if we are to live up to Professor Perry's expectation of us. We have a problem — which is not just a local one — and I have therefore chosen as my subject: 'The Evolution of Medical Societies in Britain — have they a future?'

The birth of true medical societies did not take place in Britain; they developed as the natural offspring of scientific societies formed in Italy and Germany in the 16th and 17th centuries.[2] The Renaissance, beginning in the 15th century, was primarily a rediscovery of art and literature but, as has been pointed out, 'it was inevitable that sooner or later freedom of thought and enquiry would upset that very canon of revealed truth which the Renaissance was so excited to have found.'[3] It was the gradual emergence of the spirit of enquiry, replacing the unquestioned acceptance of the Galen authority, that led medical men to come together to exchange ideas, to dissect for themselves the human body and to conduct with each other the early experiments; later there was also a need to safeguard professional standards and ethics. These objectives cannot be achieved by doctors in isolation but only when they communicate in groups or form societies. Medical schools, of a sort, had existed in Europe for four hundred years before the Renaissance — the first having begun in Salerno in 1096 — and, on the basis of what we nowadays call 'centres of excellence', it appears that 'the torch passed in succession to Montpelier, Bologna, Paris, Padua, Leyden and thence to Edinburgh.'[4] It is in Edinburgh in the early 18th century that my story opens.

Although instruction, particularly in the surgical craft, had been given in Edinburgh for two hundred years, it was John Monro, a student of the medical school of Leyden (where he had come under the influence of the great Boerhaave), who carried this torch on its last lap. I call it the last lap because, although Edinburgh was Britain's first organised medical school, 'it was last in the line of the few world-famous centres which had, in their turn, provided the only real medical training then available.'[4] John Monro's ambitions included educating his own son, Alexander, so as to befit him for the chair of anatomy and he was duly made a professor in 1720 at the age of 22. Within six years, five further professors were appointed — in physiology, chemistry, materia medica, the practice of physic, and midwifery — but it is Alexander Monro *primus* who is regarded as the founder of the Edinburgh Medical School in 1726. He was to be followed by Alexander Monro *secundus* and he by Alexander Monro *tertius.* This was an example of nepotism in medicine which Newman, in his history of medical education in the next century, has defended as not without its benefits.[5]

The hospital providing the clinical material for the Edinburgh medical faculty appears originally to have had only four beds, yet I can find no reference to the existence of a waiting list. However, building was started in 1738 on a new Infirmary and this was to have 228 beds. I mention this, partly to highlight the provision of hospital services in a capital city at that time, but also to stress the fact, that, four years before the laying of this Infirmary foundation stone, another kind of foundation was laid by six of Monro's students who, in 1734, started a society which was to become the oldest student medical society in Britain and which still exists today as the Royal Medical Society of Edinburgh.

Early in August 1734 a student by the name of Russell bought a young woman. This of course was

*The substance of the presidential address given on 10 October 1979. Because of late publication of the Journal its cover date antecedes the giving of this address.

not the first time in history that a young woman's body had been for sale — but, in this case, there was nothing shameful in the deal because the young woman was dead: she had died of ten days' fever. Russell, with five of his colleagues, went to see the Professor of Anatomy to seek permission to use the anatomy theatre for dissection. This being granted, they worked on the body for about three weeks. As at that time no preservatives were used it must, in August, have been a particularly noisome business. It was for that reason that the hospital medical schools, even into the 19th century, held their anatomy sessions in the winter months. When their dissection was finished, these six students spent a social evening at a tavern and after supper one of them, Archibald Taylor, proposed that they met fortnightly at each other's lodgings when one of them would be primed to give a dissertation in Latin or English on some medical subject chosen by their colleagues[6] who would later discuss and criticise the views expressed. A year later, only one of the six remained but he, George Cleghorn, with the newly-arrived John Fothergill and William Cullen kept the meetings going. Two years later, in 1737, the Society was formally constituted. The same year incidentally saw the beginnings of our Infirmary here in Bristol.

William Cullen must have provided a great stimulus to this embryo society. Before arriving in Edinburgh he had already studied medicine in Glasgow, been apprenticed to a surgeon there, to an apothecary in London and had gained further experience as a ship's surgeon. He eventually returned to Glasgow and is regarded as the chief founder of its medical school, being made Regius Professor of Medicine in 1751. Four years later he returned yet again to Edinburgh and, after holding chairs in chemistry and physiology, became Professor of Medicine in 1773 at the age of 63. He had an immense influence on the Edinburgh faculty. In furthering the introduction of the clinical method he became one of the most distinguished teachers of the 18th century. He encouraged his students to think for themselves but his main weakness is said to have been that he failed to exploit the experimental method and clinical research started by his predecessor, Robert Whytt — described as 'the first neurologist'.[7] Cullen was cast in the same mould as Boerhaave and 'tried to fill the deficiencies of contemporary physiology and pathology by speculation.'[7] Perhaps he would have done better if he had adopted the same attitude as did John Hunter to Edward Jenner — 'why think, why not try the experiment?' But before adopting a censorious attitude towards Cullen, his critics should remember that he lived a hundred years before Claude Bernard and stood near the great watershed of the late 18th and early 19th centuries. That watershed separated the old medicine, based on the disputation of earlier writings, requiring no contact with individual patients, from the new medicine which centred on the patient, correlating what it found from fairly crude clinical examination in life with the more revealing findings on the mortuary table. It was the challenge of this correlation, together with the advances being made in science as a whole, that provided the great stimulus to medical research in all its specialised fields. Preceding this revolution in medicine, there occurred the great social revolutions: within the span of only twenty-five years the industrial revolution in Britain, the American War of Independence and the French Revolution were to force widespread political changes.

The Medical Society of Edinburgh went from strength to strength receiving its Royal Charter from George III in 1778. We can be proud that of the four doctors mentioned in the Charter, described as Presidents of the Society, two are from our own west country — one, James Melliar of Taunton, and the other Caleb Hillier Parry of Cirencester, who, as a life-long friend of Jenner, practised as an eminent physician in Bath and described angina pectoris, hyperthyroidism and progressive facial hemiatrophy.

The supremacy of Edinburgh continued throughout the rest of the 18th century but in the first half of the 19th century the London medical schools began to evolve. It would take too long to even list the many famous names that came out of Edinburgh and who were members of its Medical Society. As Guthrie has maintained: 'Medical knowledge has long been one of Scotland's principal exports' and he quotes Dr. Samuel Johnson's observation that 'the noblest prospect which a Scotchman ever sees is the highroad that leads him to England'.[8] Certainly the influence of Scottish graduates, including some who had gone there from this country, gradually began to permeate London and later the provinces and it is important to realise that the Scottish legacy pervaded medical education and practice in England almost entirely through these young schools attached to the large teaching hospitals and through the activities of medical societies many of which were created by Scottish graduates. The English university faculties of medicine, which at that time meant Oxford and Cambridge, continued to live in the past — as well as restricting entrance to members of the Church of England. Positions here also were later reversed and by the middle of the 19th century 'when medical education in Edinburgh was in the doldrums . . . medical examinations in Cambridge were beginning to have quite a modern appearance.'[9]

In London, Guy's led the field in founding its 'Physical Society' in 1771 — including in its membership the staff from both St. Thomas's and Guy's (then known as the 'United Hospitals') as well as local practitioners and other London consultants. Two years later John Coakley Lettsom launched the Medical Society of London and to this I shall return. The next three decades saw the founding of societies at the Middlesex Hospital, in Colchester, Exeter, Aberdeen and Plymouth, at St. Bartholomew's Hospital, and in Leicester and Glasgow. I must also mention here the short-lived Gloucestershire or 'Fleece Inn Medical Society' in nearby Rodborough organised in 1788 by Jenner and Caleb Parry, the former addressing its members on cowpox vaccination and the latter on angina. 'The society was also known as the local Medico-Convivial Society in distinction from another local society the Convivio-Medical Society'[10] founded twenty years earlier by Jenner, with the help of Ludlow of Sodbury and Fewster of Thornbury, and which had met at the Ship Inn at Alveston.[11] It was ironically the members of this earlier society who 'complained that they found Jenner's scientific papers tedious.'[10] If only those local doctors had known how uniquely privileged they were. The book of minutes of the Fleece Society is treasured by the Royal College of Physicians in London.[12] Because these early societies attracted doctors from rural surroundings some of them held their meetings monthly, near the time of the full moon, to render their members' homeward journey less hazardous. This was certainly the case in Plymouth and Norwich. The Lunar Society founded in Birmingham in 1766 was so named because of the time of its meetings; it was not primarily a medical society but Erasmus Darwin (Charles' grandfather) and William Withering were two of the distinguished doctors in this select circle.[13]

Of these early societies pride of place and certainly of influence goes to the Medical Society of London. At the time of its founding the physicians, the surgeons and the apothecaries were in open rivalry and it says much for the diplomacy of Lettsom, a Quaker, who at the age of twenty-nine managed to draw together thirty physicians, thirty surgeons and thirty apothecaries. The Council of the Society also preserved this parity of representation being made up of three physicians, three surgeons and three apothecaries. His kindly personality has been contrasted with 'the brusque tradition handed on from John Radcliffe.'[14] In 1773, as well as founding the Medical Society of London, Lettsom was elected a Fellow of the Royal Society. It is said that he used to see about fifty patients before breakfast and afterwards visited his paying patients some of whom were wealthy city merchants. If this speed of working shocks us, we must remember that detailed physical examination of the patient had not yet been developed. Apart from taking a history, physicians did little more than observe the face, including a look at the tongue, feel the pulse, look at the urine, the blood after letting and, if they were really keen, the faeces. Lettsom had no stethoscope: Laennec did not invent it until four years after his death. At the height of his career his income is said to have been £12,000 a year (well over £130,000 by today's standards). He married a wealthy heiress and they lived in the grand style with their children at Grove Hall in Camberwell — an estate which spread over ten acres. Nevertheless, he was extremely generous in his gifts to individuals and donations to institutions and in 1787 he presented the Medical Society of London with premises under a trust at 3 Bolt Court in Fleet Street.

Lettsom's many other accomplishments give some idea of the social conscience as well as the prodigious energy of this remarkable man. He was the first to use the Dispensaries for teaching students; he also visited the poor in their own homes — then almost unheard-of amongst physicians. The conditions he found as a prison doctor appalled him and he made some early and astute observations on typhus. He and Fothergill were close friends of Dr. John Howard the great pioneer of prison reform. Although initially sceptical of Jenner's theory on cowpox inoculation, Lettsom later supported it and sent a copy of Jenner's first pamphlet to his friend Waterhouse, Professor of Medicine at what became Harvard, which led to the acceptance of Jennerian vaccination throughout America. The Medical Society of London indeed claims that 'there is no greater honour to our Society than the part it played in the development of American medicine. Lettsom himself held sixteen American honours and amongst the 56 signatories of the Declaration of Independence in 1776 there were six physicians.'[15]

In 1786 Dr. James Sims became President of the Medical Society of London and, although the Society continued to flourish and exerted considerable influence in both medical and social spheres, he was one of those unfortunate men who clung to power and remained President for 22 years. After 19 years of his presidency, in 1805, some members rebelled and formed themselves into the Medical and Chirurgical Society which grew to a membership of nearly 2000 by the 1850s. By this time however specialist societies were making their appearance and the (by now Royal) Medical and Chirurgical Society began to suffer a fall in membership. Once again there was a threat that the medical profession might become fragmented and several attempts (including one by the Medical Society of London) were made to achieve some form of amalgamation. Yet it was not until 1907 that the Royal Society of Medicine was finally formed of 17 specialist societies. Its subsequent history and influence is well known and it still fulfils a useful role — now comprising 34 sections — just double the original number. It is significant and sad that fifty years were to pass before a section of General Practice was formed.

Going back to the late 18th century we find that many an early society started as a medical book club in which members subscribed to buy books, circulated them amongst colleagues and later had the opportunity of either choosing which copies they themselves wanted to keep or adding them to the society's library. This was often housed in the local infirmary. Records are scarce and research has often had to rely on the inside covers of old medical books where the circulation slips give valuable information. After Liverpool (1770), Bristol was in the vanguard of this movement, a society having been founded in 1788. By today's standards, subscriptions were costly, rules strict and fines heavy. The entrance fee to the Birmingham Medical Library in 1825 was ten guineas and the annual subscription one guinea. At this price it is perhaps not surprising that, when this library was dissolved, one member refused to surrender his books and a duel between him and the representative of the library was only prevented at literally the last minute.[16] Many of the book clubs also held discussion meetings, not to mention an annual dinner. At one of these, in Sheffield in 1834, no less that 21 toasts were proposed. The Lancaster Medical Book Club (founded in 1823) is still very much alive. Some societies bought not only books but, as they came into being, medical journals as well. A few produced journals themselves but only four, including our own, continue to do so.

Throughout the 19th century local medical societies of a general character mushroomed all over Britain. In a recent study, Poynter found that the Medical Directory of 1868 listed 82 'authentic' societies; 50 of these are no longer in existence. He used the word 'authentic' because he found the total list included societies such as the Cotswold Naturalists' Field Club — which is noteworthy for 'the light which this throws on the leisure interests of medical men a century ago, when they still had leisure to indulge them.'[17]

As well as their educational and social functions, medical societies played a considerable part in formulating the ideas which led, after almost endless controversy and feuds, to the legislation governing medical education and ethical standards.[18] Charles Hastings of Worcester founded the Provincial Medical and Surgical Association in 1832 from which the British Medical Association arose in 1856; the Midland Medical and Surgical Reporter which he edited (from 1828) was the forerunner of the BMJ.

It was a period of intense creative thinking and saw the beginnings of true scientific medicine whose growing points it was important to recognise and follow. In those times it was still possible to keep up to date — if only just. Keeping up to date gradually became a losing battle. Being well-informed of the rapidly increasing changes in the whole field of medicine very soon became a battle lost. Accepting defeat, specialisation was inevitable and with it the rise of specialist associations — local, national and international. Rushing headlong into the 20th century the branch of medicine to fall hindmost was general practice and, as if to fulfil the saying 'from him that hath not shall be taken away even that which he hath', the introduction of the National Health Service in 1948 made hospitals its first priority. This ensured a rising standard of hospital practice but had a depressing effect on the morale of family doctors who suddenly found themselves ill-equipped to cope with the ever-increasing demands made upon them. Worse still, the depressive illness of general practice developed unmistakable paranoid features so that some of the profession's self-inflicted wounds, which Lettsom and his friends had done their best to heal, threatened to reopen.

In 1961, a conference organised by the Nuffield Provincial Hospitals Trust was held in Christ Church College Oxford with its Regius Professor of Medicine,

Sir George Pickering, in the chair. What has been called the 'Postgraduate Centre Movement' grew out of this, the aim being to provide educational facilities in all district general hospitals for junior hospital staff and for local general practitioners.[19] It was also hoped to improve communication between all hospital staff and doctors working in the community — restoring the relationship which the NHS had impoverished. Postgraduate medical centres sprang up at a much faster rate and in greater numbers than had the medical societies in their time and there are now 339 such centres in England and Wales.

It was the founding of specialist associations and new colleges, together with the surfeit of postgraduate meetings provided in the new centres, that were two of the most significant factors which insidiously undermined the place of the traditional medical societies. Wives and a very proper recognition of the claims of family life came a close third. Membership in many places fell and actual attendance at meetings dropped even further. Many societies ceased to exist.

Is there any point in us continuing to duplicate the dissemination of medical knowledge? With organised continuing education, family doctors are catered for — though a move towards more self-catering is already afoot as general practice strives to become a discipline in its own responsibility. It is sometimes said that consultants find our meetings valuable in acquainting them with the advances in specialties other than their own. In fact, what often happens is that a specialist speaker is supported more by the attendance of colleagues in his own specialty and, except for 'the regulars', it is frequently disappointing how little apparent interest is shown by colleagues from other disciplines.

In order to find out what is happening to other medical societies which still survive I wrote to the honorary secretaries of the 72 societies appearing in the Medical Directory and also to those of Oxford and Cambridge which for some reason do not appear therein. I excluded the many specialist and other associations not relevant to the enquiry; likewise I did not write to the eponymous societies many of which are primarily dining clubs. The overall response was 73%. This in itself was encouraging and underlined the fact that, as here in Bristol, the life-force of the societies is the energy and enthusiasm of their honorary secretaries.

The questionnaire is reproduced in Table 1. My accompanying letter explained that I was making a study of the history of medical societies and was concerned about their future role.

The years of founding stretched from 1737 to 1967. Present memberships varied from 38 to 450 in centres without a medical school and from 160 to 695 in those with a school; Manchester Medical Society is exceptional, being a sort of provincial RSM with a membership of 1,829 and holding 64 meetings a year. The average attendance at meetings is less relevant than the range which, taking all societies, varied from 15 to 150 but an eminent speaker — in one case an ecclesiastic — could draw as many as 400. The number of meetings a year varied from 2 (which were purely social gatherings) to 16. The centre with the smallest membership had 18 meetings a year but this was in a rural area so that the medical society was still acting as its main postgraduate activity. The number of meetings on non-medical subjects varied from none to all.

The rest of the statistics are summarised in Table 2.

The most significant but anticipated finding was the admission that the competition of organised postgraduate education had forced a change of emphasis and that this change had not been towards a greater interest in social problems allied to medicine but towards social activities allied to doctors. Of the various sporting events which are traditionally organised, golf was the most popular, being named by at least nine societies.

Question 11 drew forth a great variety of comments. Most of the secretaries saw the future role as a continuing common forum for all branches of medicine dealing with general rather than specialist subjects. Particular emphasis was laid on the need to bring together those who work in hospitals with those outside. Reading between the lines, one could discern that some medical centres had not achieved this part of their function as well as they might have done. The situation varies much from place to place but it was clear that what brings doctors together most effectively is the social event to which spouses and other guests are invited. This is also a means of welcoming newcomers to the area. Two secretaries boldly stated that their societies existed 'to entertain and inform — in that order'; some saw a useful paramedical trend in 'filling gaps' in the medical scene. Several societies appreciated the need to constantly rethink their role and two of them were actually holding a referendum on their future.

Only one society stood out as having 'a strong tradition of literary and historical papers as well as an interest in ethical and social problems.' One society still excludes women doctors from membership. In the evolution of medical societies a fairly typical story was the rejection of the application of a woman doctor in 1906 to become 'a subscriber to the library of the Leicester Medical Society as the only woman doctor in the town.' Thirteen years later, after the First World War, the council recommended the admission of women practitioners and one of the two then elected became their first woman president in 1935. The Cambridge Medical Society now accords life-membership to the spouses of deceased colleagues. The York Medical Society must be the envy of many in that it boasts a Georgian house, having started as a wine society with its own cellars.

It appears therefore that many of our surviving societies in Britain have become convivio-medical —

**TABLE 1**

1. Present name of Society ..........
2. Year of founding ..........
3. Present number of members ..........
4. Average attendance at meetings ..........
5. Is this decreasing/steady/increasing? ..........
6. Do meetings mainly attract older doctors? ..........
7. Number of meetings a year ..........
8. Number of these meetings (if any) on non-medical subjects ..........
9. TRENDS (a) Is there a trend towards papers or discussions on ethical/social problems rather than clinical subjects?

   ..........

   ..........

   (b) Is there any other trend, e.g. literary, historical, philosophical papers, symposia or debates, social activities?

   ..........

   ..........
10. Do you publish a Journal? ..........
11. What do you *personally* feel is the future role of Medical Societies such as yours?

    ..........

    ..........

**TABLE 2**

| | Centres with Medical School excl. London | Centres without Medical School | Primarily Student Societies in London | TOTALS |
|---|---|---|---|---|
| Societies approached | 15 | 51 | 8 | 74 |
| Percentage response | 87 | 69 | 62 | 73 |
| ANSWERS TO QUESTIONS 5, 6, 9(a) and (b) and 10 — EXPRESSED AS PERCENTAGES | | | | |
| Q. 5. Meeting attendance: | | | | |
| decreasing | 23.0 | 25.7 | | 25 |
| steady | 38.5 | 51.4 | | 48 |
| increasing | 38.5 | 22.9 | | 27 |
| Q. 6. Attracting older doctors | 54 | 37 | Replies not relevant to enquiry | 37 |
| Q. 9. (a) Trend towards ethical/social problems | 54 | 14 | | 23 |
| (b) Other trends: | | | | |
| literary | 15 | 11 | | 11 |
| historical | 30 | 17 | | 19 |
| philosophical | 15 | 8 | | 9 |
| social activities | 23 | 54 | | 41 |
| Q.10. Journal published | 19 | 0 | | 4 |

and some entirely convivial. I am far from depreciating the social and professional dividends which accrue from relaxed conviviality. Such meetings, laced with food and drink can bring people together, increase mutual understanding and reduce personality clashes more effectively than any earnest meeting.

Hard on the heels of the 1961 Christ Church Oxford Conference which heralded the postgraduate centre movement, another movement arose two years later. History was being repeated. Once again students took the initiative, though not this time in Edinburgh but in London where in 1963 the London Medical Group was formed. Students with a strong social awareness began to question the ethics of the profession they were about to enter. They realised that advances in medical technology, together with changes in social values, raised important moral issues which had to be faced. What better time to start thinking about these issues than when still a student — before the pressures of day-to-day work begin to overwhelm and before the personal responsibilities of clinical decisions begin to agonise? Edinburgh was quick to follow the London example in 1967 and so was Newcastle. By 1972 the original London students had qualified and, as junior doctors, they founded the Society for the Study of Medical Ethics. Since then student Medical Groups have been formed in Sheffield, Glasgow, Birmingham, Manchester, Liverpool, Aberdeen, Southampton, Dundee, Cambridge, Cardiff and here in Bristol.

The governing body of the Society for the Study of Medical Ethics and the Editorial Board of its Journal include not only respected leaders of our profession but also people eminent in other walks of life. The strength of this movement lies in its organisational independence, its multidisciplinary basis and its non-partisan approach with a refusal to be dominated by pressure groups. It seeks to 'influence the quality of both professional and public discussion of medico-moral problems; . . . to ensure a high academic standard for this developing subject; (and) to stimulate research in specific problems.'[20] In this latter context, research fellows were appointed in 1975 by the Edinburgh Medical Group in conjunction with the University there.

In London now the Medical Group organises open lectures and symposia every fortnight from October to May and these are held in the twelve London teaching hospitals. I need not dwell on the activities of our own Bristol Group whose Consultative Council is chaired by Dr. Ian Bailey, a former honorary secretary of our Society. I will, however, quote at random a few of the titles of recent London meetings and of articles in the Journal of Medical Ethics: 'A new ethical approach to abortion and its implications for the euthanasia dispute' — 'Marital breakdown in the light of changing sexual attitudes' — 'Who runs the place anyway? The role of unions and doctors in the NHS' — 'The alcoholic: someone who drinks more than his doctor?' — 'Doctors, prisoners and the State — a conflict of interest?' — 'Atomic power: is the health hazard too great?' — 'Should striking doctors be struck off?' — 'Does the end justify the expense?' — 'Confidentiality — an anachronism in modern medicine?' — and so on. Such subjects may not sound as if they would turn us on at the end of a busy day, but do they at all times turn us off? Do we turn away because we know from experience that discussion of our ethical dilemmas so often leads to deepening disagreement rather than to consensus? Are we then never to discuss ethical problems?

There are certain dichotomies which seem to impede our progress along this road. One of these dichotomies is political — the divide between 'right' and 'left'; another is religious — the gulf between theists and atheists. There are also deep differences among theists: between catholics and protestants, not to mention the disagreements among dissenters. Many doctors give serious thought over the years to their position in these matters, yet it is often surprising how we, who have to cope with so much uncertainty in our work, adopt such inflexible attitudes in other areas of our lives. After years of preoccupation with medicine we seem to possess neither an historical perspective nor a philosophical base from which to proceed. Is it any longer acceptable to remain so compassless? Is it even responsible? Will not our successors stand amazed at our apparent lack of concern for the great holes in the ethical fabric of our profession? Professor Basil Mitchell in an essay entitled: 'Is a moral consensus in medical ethics possible?' has this to say: '. . . the habit of fair and sympathetic scrutiny of the opposing positions will at least ensure that those positions are held by their adherents in their most defensible forms and not in a blindly partisan fashion.'[21] I believe that the surviving medical societies would fulfil a really valuable function if they accepted this challenge. In this context, symposia are much better than debates: the effect of debate (so beloved of politicians) is to polarise, while the purpose of symposia should be to work towards agreement.

There is another and not unconnected role that we could play and that is in fostering the revival of culture in medicine. We do well to remember that the early physicians were primarily men of learning in the broad sense; they knew virtually nothing of the science of medicine and throughout our history, from the men of eminence to those in the obscurity of rural practice, many of our best doctors had a strong cultural background.

Lord Moran once wrote: 'When culture has gone from the leaders of our calling, we shall no longer remain a profession.'[22] Speaking as one whose education and training have been primarily scientific, I would have stressed the need for a new cultural emphasis on behalf of everyone of us. This is certainly the view of Pickering whose Nuffield Lecture to the Royal Society of Medicine in 1976 was entitled: 'Medicine at the Crossroads: Learned Profession or Technological Trades Union?'[23]

Is there no answer to the problem of early specialisation, beginning as it now does with children at school? There may be no easy answer to the selection of medical students but this should not prevent us reflecting on the price we pay for an educational system which would exclude Charles Darwin and James Mackenzie from even entering a modern medical school. Both these were men of culture and original thought, yet Darwin was regarded as too sensitive and academically lazy and Mackenzie's mother was told: '. . . your James is the most stupid boy in the school.'[24] It is often argued — and with some justification — that many school-children, especially those who are interested in science, may develop an antipathy to literature by being forced to study Shakespeare and poetry at an age when they do not appreciate their value. This should not prevent us later — when we have passed out professional examinations — from spending some of our time catching up on what we have missed. Some doctors already do this. We are fortunate today in the opportunities provided by radio and television, by local lectures and by the great outpouring of books synthesising for the layman the result of research in history and the humanities. We are

*Continued on page 13*

*Continued from page 10*
particularly fortunate in Bristol with our theatres.

The best writers of novels, plays and poetry have an insight into people and their dilemmas which often puts us, as doctors, to shame. Our education in these matters has been dominated by the analytical approach of the behavioural scientists and the sociologists. On the one hand we have George Eliot's description of the insecure child:

'A child forsaken, waking suddenly,
Whose gaze afeared on all things round doth rove,
And seeth only that it cannot see
The meeting eyes of love.'[25]

On the other, we have our clinical summary: 'this child's enuresis and antisocial behaviour are the result of prolonged parental disharmony, depriving it of affection and preventing it from developing a satisfying identity in the family situation and in the school context.' I am not suggesting that our case notes should be written in verse, full of emotive, inexact and sentimental phrases. What I maintain is that an acquaintance with our literature, in the form of the novel, the play, the poem or the biography, may give us more insight and therefore make us more effective in what we do for our patients.

To quote a recent editorial on this subject: 'Another consequence of producing doctors and other scientists who have virtually no knowledge of history, philosophy or literature is that their ignorance limits their horizons and may blind them even in their own discipline . . . After half a century of ever-increasing emphasis on scientific knowledge, are the physicians of the future beginning to refresh their minds from the classics?'[26] I am persuaded that placing a new emphasis on the cultural aspect of life would greatly benefit us and therefore our patients. It would make us better doctors in our consultations, wiser doctors in finding solutions to our ethical dilemmas and more perceptive doctors in assessing priorities and maintaining a healthy perspective.

In this whistle-stop journey, covering the last 250 years, I have tried to show: firstly, how the early medical societies fulfilled, in their time, the needs of our profession in spreading the new knowledge, as well as in building up its ethical standards in a climate which favoured fellowship and loyalty and which gradually overcame the old feuds; secondly, how our changing needs required a purposeful organisation of postgraduate training and continuing education in medical centres; thirdly, how our surviving societies have reacted; fourthly, how our needs are again changing in finding answers to moral problems which yearly become more pressing and more difficult in clinical practice and, indeed, in the research influencing that practice. Finally, I have put forward another need which may underlie all others — the need for a revival of culture in medicine.

My own feeling is that, as medical societies, we should further reduce the number of meetings devoted to purely scientific subjects since these tend to duplicate the function of the postgraduate centres. Instead we should establish a forum where we can broaden our learning and thereby compensate for its restrictiveness in our early years. Such a professional renaissance, which would embrace the frank and informed consideration of ethical problems, could have a far-reaching effect. It would enable us, as a profession, to arrive at some degree of consensus which hopefully could influence the decisions made in the name of the people by politicians in the guise of democracy. In pursuing this goal I do not invisage medical societies becoming elitist clubs holding terribly earnest gatherings. As well as serious discussion there must continue to be a place for a lighthearted talk and the occasional social event. Just as Moran thought we were doomed without culture, so I believe we are doomed if we lose our sense of humour — especially the ability to laugh at ourselves.

I wish the Society well — but, as to its future, the secret of survival is change.

### ACKNOWLEDGEMENTS

My thanks are due to the fifty-five honorary secretaries of Medical Societies throughout Britain who not only answered my questions but, in many cases, wrote personal letters expressing interest and encouragement, enclosing brief histories of their societies and lists of their current programmes. I confess my indebtedness to the many sources I have consulted and especially to Dr. Anthony Batty Shaw for his diligent research on 'The Oldest Medical Societies in Great Britain'. I am also grateful to Mr. Brian Jones, our Medical School Librarian, who traced the books I still needed.

### REFERENCES

1. PERRY, C. B., *Bristol Medico-Chirurgical Jl*, 1976, *89* (iii) 30.
2. THORNTON, J. L., Medical Books, Libraries and Collectors, 1966 A. Deutsch, 194—5.

3. NEWMAN, C., The Influence of Medical Education on the Evolution of Medical Practice in Britain, *The Evolution of Medical Practice in Britain,* Ed.Poynter, F.N.L., Pitman Medical, 1961, 26.
4. MASSON, A. H. B., The Edinburgh Medical School, *History of Medicine,* Quarterly Winter 1972/3, 3.
5. NEWMAN, C., The Evolution of Medical Education in the 19th cent., Oxford, 1957, 143.
6. GRAY, J., History of the Royal Medical Society, Ed. Guthrie, D., Edin. U.P., 1952, 15—16.
7. Ibid, 33.
8. GUTHRIE, D., Scottish Influence on the Evolution of British Medicine, *The Evolution of Medical Practice in Britain,* Ed. Poynter, F.N.L., Pitman Medical, 1961, 145.
9. NEWMAN, C., The Evolution of Medical Education in the 19th cent., Oxford, 1957, 104 and 110.
10. BATTY SHAW, A., The Oldest Medical Societies in Great Britain, *Medical History,* 1968, Vol.XII No.3, 234.
11. BISHOP, W. J., Medical Book Societies in England, *Bulletin of the Medical Library Association,* 1957, *45,* 338.
12. THORTON, J. L., Medical Books, Libraries and Collectors, 1966 A. Deutsch, 204.
13. GILL, C., History of Birmingham, Oxford U.P., 1952, Vol.I, 135—6.
14. HUNT, T., Dr. Lettsom and the Foundation of the Society, *The Medical Society of London, 1773—1973,* Ed. Hunt, T., Heinemann, 1972, 2.
15. JEPSON, E. M., The Society's Influence on Medicine and the Community, *The Medical Society of London, 1773—1973,* Ed. Hunt, T., Heinemann, 1972, 37.
16. BISHOP, W. J., Medical Book Societies in England, *Bulletin of the Medical Library Association,* 1957, *45,* 344.
17. POYNTER, F. N. L., British Medical Societies, 1868—1968, *The Practitioner,* 1968, *201,* No.1202, 238.
18. McMENEMEY, W. H., The Influence of Medical Societies on the Development of Medical Practice in Britain, *The Evolution of Medical Practice in Britain,* Ed. Poynter, F.N.L., Pitman Medical, 1961, 67.
19. LISTER, J., The Postgraduate Centre Movement, *Update,* October 1978, 43.
20. SHOTTER, Prebendary E. F., (personal communication).
21. MITCHELL, B., *Journal of medical ethics,* 1976, *2,* 23.
22. NEWMAN, C., The Evolution of Medical Education in the 19th cent., Oxford, 1957, 51.
23. PICKERING, G., *Proceedings of the Royal Society of Medicine, 70,* (1), 16.
24. MAIR, A., Sir James Mackenzie, MD, 1853—1925, General Practitioner, Edin. & London, Churchill Livingstone, 1973, 8.
25. ELIOT, George, Middlemarch (Edited W. J. Harvey) Penguin English Library, 224.
26. EDITORIAL, *Jl of The Royal College of General Practitioners,* 1976, *26,* 169.

